# Cyclin D mediates tolerance of genome-doubling in cancers with functional p53

**DOI:** 10.1093/annonc/mdw612

**Published:** 2016-11-17

**Authors:** A. Crockford, L. P. Zalmas, E. Grönroos, S. M. Dewhurst, N. McGranahan, M. E. Cuomo, V. Encheva, A. P. Snijders, J. Begum, S. Purewal, J. Cerveira, H. Patel, M. J. Renshaw, C. Swanton

**Affiliations:** Translational Cancer Therapeutics Laboratory, The Francis Crick Institute, London, UK

**Keywords:** chromosomal instability, tetraploidy, p53, cyclin D, p21

## Abstract

**Background:**

Aneuploidy and chromosomal instability (CIN) are common features of human malignancy that fuel genetic heterogeneity. Although tolerance to tetraploidization, an intermediate state that further exacerbates CIN, is frequently mediated by *TP53* dysfunction, we find that some genome-doubled tumours retain wild-type *TP53*. We sought to understand how tetraploid cells with a functional p53/p21-axis tolerate genome-doubling events.

**Methods:**

We performed quantitative proteomics in a diploid/tetraploid pair within a system of multiple independently derived *TP53* wild-type tetraploid clones arising spontaneously from a diploid progenitor. We characterized adapted and acute tetraploidization in a variety of flow cytometry and biochemical assays and tested our findings against human tumours through bioinformatics analysis of the TCGA dataset.

**Results:**

Cyclin D1 was found to be specifically overexpressed in early but not late passage tetraploid clones, and this overexpression was sufficient to promote tolerance to spontaneous and pharmacologically induced tetraploidy. We provide evidence that this role extends to D-type cyclins and their overexpression confers specific proliferative advantage to tetraploid cells. We demonstrate that tetraploid clones exhibit elevated levels of functional p53 and p21 but override the p53/p21 checkpoint by elevated expression of cyclin D1, via a stoichiometry-dependent and CDK activity-independent mechanism. Tetraploid cells do not exhibit increased sensitivity to abemaciclib, suggesting that cyclin D-overexpressing tumours might not be specifically amenable to treatment with CDK4/6 inhibitors.

**Conclusions:**

Our study suggests that D-type cyclin overexpression is an acute event, permissive for rapid adaptation to a genome-doubled state in *TP53* wild-type tumours and that its overexpression is dispensable in later stages of tumour progression.

Key messages

Genome-doubling is associated with tumourigenesis, poor prognosis and drug resistance. Our study provides functional insight into the early mechanisms of tetraploidy tolerance in *TP53* wild-type tumours, describing a central role for D-type cyclins in overcoming p53-mediated G1 arrest and allowing tolerance to tetraploidy.

## Introduction

Despite significant advances in the management of human cancers over the past 20 years, the majority of patients with metastatic disease or tumours not amenable to surgical resection remain incurable. Intratumour heterogeneity (ITH) contributes significantly to this unsatisfactory outcome [1].

ITH can be generated by chromosomal instability (CIN), which is characterized by an elevated rate of karyotypic change through numerical and structural chromosomal defects. CIN is accompanied by a tolerance mechanism, such as loss of *TP53*, and an increase in chromosome segregation error rate resulting in aneuploidy, a state of abnormal chromosome number [2].

Tetraploidy is a specific state characterized by genome-doubling and has been proposed to be an aneuploidy intermediate [3]. It has been demonstrated that tetraploid cells can be generated by mitotic slippage or cytokinesis failure and as mammalian cells have evolved systems to abrogate the proliferation of tetraploid cells, these cells normally arrest in the G1 phase of the cell cycle [4, 5].

In human tumours, *TP53* mutations have been shown to correlate with polyploidy or tetraploidy, highlighting its integral role in the tetraploidy checkpoint [6, 7]. *TP53*^*−*^^*/*^^*−*^ tetraploid, but not diploid, cells generated through cytokinesis failure have been shown to form tumours that exhibit an array of chromosomal abnormalities, suggesting that tetraploidy is highly tumourigenic [8]. Previous work from our laboratory has shown that spontaneously arising, *TP53* wild-type, HCT116 tetraploid clones tolerate segregation errors better than diploid clones and are subject to increased CIN over time in culture [9]. Understanding how tetraploidy and chromosome segregation errors are tolerated in cells with a functional p53 axis could provide opportunities for therapeutic intervention to limit cancer diversity, adaptation and evolution.

In this study, we report that D-type cyclins can override the p53/p21-dependent checkpoint in tetraploid cells and that *TP53* wild-type tumours associate with increased expression levels of D-type cyclins. Importantly, we provide evidence that cyclin D-overexpressing cells do not show enhanced sensitivity to CDK4/6 inhibition and thus question their therapeutic potential in targeting cyclin D-overexpressing tumours.

## Materials and methods

### Cell culture

HCT116 and RPE-1 cells were obtained and authenticated by STR profiling with 16 STS markers, by Cell Services at the Francis CRICK Institute, UK (see also, “Supplementary Materials and Methods”, available at *Annals of Oncology* online). Parental cell lines and their derivatives were grown in Dulbecco’s Modified Eagle Medium supplemented with 10% Foetal Bovine Serum and 1/10 000 units penicillin/streptomycin (Sigma–Aldrich) at 37**°**C in a 5% CO_2_ atmosphere.

### SILAC

DC14 and TC13 (passage five and 42) were cultured in DMEM supplemented with 150 mg/l L-Proline (Sigma–Aldrich) and ‘heavy’ or ‘light’ isotopes. Each clone, at both early and late passages, was cultured in heavy or light media, as replicate experiments that could be inversely correlated after analysis. Cells were lysed and mixed at a 1:1 ratio. Next, lysates were quantified by Bradford assay before being separated by SDS–PAGE and stained with EZ blue (Sigma–Aldrich). Gel slices were prepared for mass spectrometric analysis using the Janus liquid handling system (Perkin–Elmer).

### Bionformatics analysis of TCGA data

Mutation data and segmented copy number data from TCGA were obtained from [10]. Genome doubling and wGII was estimated as previously described [9]. Pre-processed RNA-seq data, normalized using the RSEM method and summarized to gene level, were downloaded from the TCGA data portal. RNA-seq data was log2 transformed, and expression levels of *CCND1*, *CCND2*, *CCND3*, *CDKN1A*, *CDKN2A* and *TP53* were further normalized relative to expression of *TBP*. Correlation analysis was performed using a Spearman’s Rank correlation, while expression differences in *TP53* wild-type versus mutant were compared using a Wilcoxon test.

### Clonogenic assays

Clonogenic assays were performed as described [1]. Equal number of cells were seeded in the absence or presence of drug and allowed to form colonies for a minimum of 10 days. Plates were fixed in 4% PFA, washed with PBS and stained with crystal violet (0.05% w/v) in methanol (20% v/v). Plates were imaged with a flatbed scanner and either counted manually or by automated colony counting using Mathematica v10.3 (Wolfram Research). Following plate alignment, individual wells were cropped and background subtracted. Objects were segmented using automatic thresholding (Otsu’s cluster method) and touching objects separated using a watershed algorithm. Objects smaller than the expected size for a colony of 50 cells were excluded from the count.

### Statistical analysis

Statistical analysis of experiments, unless otherwise indicated, was performed by unpaired Student’s *t*-test. *P* values are indicated as follows: *NS*> 0.05, *≤ 0.05, **≤ 0.01, ***≤ 0.001).

## Results

### Spontaneously arising HCT116 tetraploid clones overexpress cyclin D1

**Figure 1.**
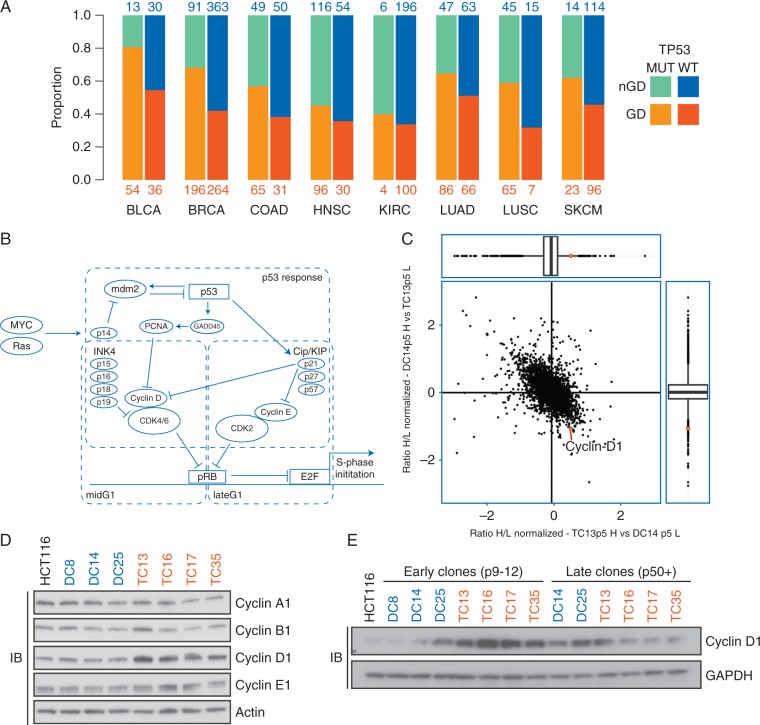
Tetraploidy tolerance in a *TP53* WT background. (A) The proportion of genome-doubled (GD) versus non-genome-doubled (nGD) tumours is indicated for each cancer type. (B) Schematic representation of major genes involved in the G1/S pathway and p53 response pathways. (C) SILAC correlation plot displaying two inversely labelled replicate experiments. Early TC13 cells were labelled ‘heavy’ (H) on the horizontal axis and ‘light’ (L) on the vertical axis, while early DC14 cells were labelled inversely. In either case, ‘heavy’ species were divided by ‘light’ and ratios representing log2 fold differences between clones were plotted. Box plots showing the median (stripe), the 25th–75th percentile (box) and outliers (open circles) for both replicate experiments. Cyclin D1 is indicated. (D) Immunoblot (IB) of all major cyclins in diploid (DC) and tetraploid clones (TC). (E) Immunoblot of cyclin D1 levels in diploid and tetraploid, early and late clones, as indicated by the passage number.

Chromosomal missegregation normally leads to activation of the p53 pathway, induction of p21 and ultimately to cell cycle arrest [2]. Consistent with p53 playing a key role in tetraploidy tolerance, an analysis of eight tumour types revealed that genome doubling is more likely to occur in *TP53* mutant than wild-type tumours [10]. Further analysis of these tumour types revealed that 47% of all genome-doubled tumours were *TP53* wild-type (Figure 1A), suggesting the existence of additional tolerance mechanisms in addition to p53 inactivation.

We have previously shown that spontaneously arising *TP53* wild-type tetraploid HCT116 clones have an increased tolerance to both CIN and segregation errors relative to diploid clones [9]. Whole exome sequencing (WES) identified no coding mutations in *TP53*, *CDKN1A or RB1*, another gene commonly mutated in tetraploid cells [11]. When investigating the TCGA dataset, we identified genome-doubled tumours that were wild-type for both *TP53* and *RB1* (supplementary Figure S1a, available at *Annals of Oncology* online), similar to the isogenic HCT116 model used in this study. In fact, no mutations in any other components of the G1/S checkpoint were found to be specific to the tetraploid clones (Figure 1B).

To investigate global differences between diploid and tetraploid HCT116 clones we performed quantitative proteomics (SILAC). SILAC analysis of an early passage diploid clone (DC14) compared to a tetraploid (TC13) revealed an enrichment of several proteins in the early tetraploid clone (Figure 1C). Validation experiments showed that in the four tetraploid clones investigated, only cyclin D1 was consistently upregulated both at the protein and mRNA level, relative to the three diploid clones and parental HCT116 cells (Figure 1D and supplementary Figure S1b and c, available at *Annals of Oncology* online). This observation was specific to cyclin D1, as protein levels of cyclin A1, B1 and E1 remained unchanged between all clones (Figure 1D).

The elevation of cyclin D1 protein levels seemed to be of a transient nature, as SILAC experiments of tetraploid clones at later passages failed to show consistently high levels of cyclin D1 (supplementary Figure S1d, available at *Annals of Oncology* online). Indeed, immunoblotting analysis confirmed that cyclin D1 protein levels were indistinguishable between late tetraploid and diploid clones (Figure 1E).

### Cyclin D-overexpression allows tetraploid tolerance after cytokinesis failure

The continuous acquisition of mutations in HCT116 cells, driven by microsatellite instability [12], has the potential to compromise the functional determination of the role of cyclin D1-overexpression in tetraploid tolerance. We therefore generated an alternative experimental system by using the retinal pigment epithelial (RPE) cell line, a stable diploid immortalized non-transformed cell line with a functional p53 response and G1/S pathway. Importantly, as RPE cells undergo G1 arrest upon pharmacological induction of tetraploidy [13], this system would demonstrate the direct effect of cyclin D1 overexpression on the tetraploidy checkpoint.

RPE cells were virally infected with vectors of the FUCCI reporter system (supplementary Figure S2a, available at *Annals of Oncology* online), providing an elegant method to distinguish between cell cycle phases, with G1 cells stained red (mCherry) and G2 cells stained green (Venus) [13, 14]. These cells were subsequently infected to stably overexpress cyclin D1 (supplementary Figure S2b, available at *Annals of Oncology* online).

**Figure 2.**
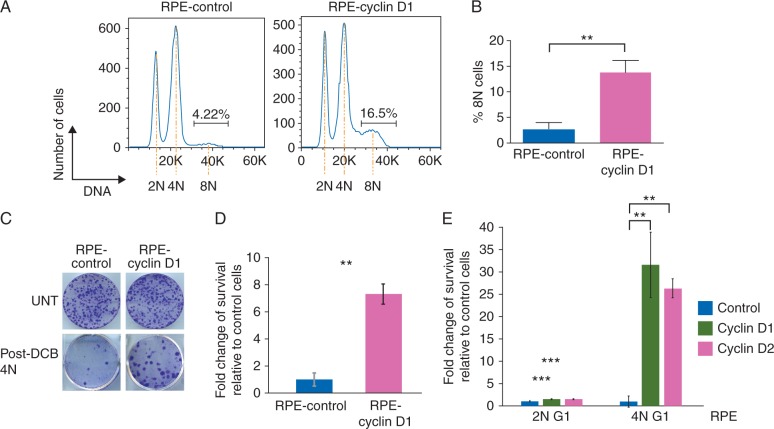
D-type cyclin-overexpression confers tetraploidy tolerance. (A) DNA ploidy profiles showing the percentage of 8N RPE-FUCCI, control or cyclin D1-overexpressing cells, post-DCB treatment (2 μM, 18 h). The basal levels of tetraploidization are expressed as percentages of the total number of cells analysed. (B) Quantification of the mean percentage of 8N cells from triplicates of three independent experiments. (C) DCB-treated, RPE-FUCCI control or cyclin D1-overexpressing tetraploid (4N) cells were plated for clonogenic assays and stained. Untreated (UNT) cells were plated for plating efficiency (PE). (D) Quantification of colony forming assays, presented as a fold change relative to RPE-FUCCI control cells. (E) Colony forming units from triplicates of two independent experiments were counted and presented as a fold change relative to RPE-FUCCI control cells.

Treatment with dihydrocytochalasin B (DCB), an inhibitor of actin polymerization and contractile ring formation [8], resulted in cytokinesis failure and formation of a tetraploid (4N) population arrested in G1, whereas cells that had escaped the checkpoint and replicated their DNA comprised an 8N peak (Figure 2A). Control cells exhibited only a small background level of 8N tetraploid cells, whereas RPE-cyclin D1 cells displayed a significant increase in the 8N population after DCB treatment (Figure 2A and B). Cyclin D1-overexpression did not increase the basal tetraploid fraction in this system (supplementary Figure S2c, available at *Annals of Oncology* online), suggesting a specific role for cyclin D1 in tetraploidy tolerance and that tetraploid cyclin D1-overexpressing cells are able to override the tetraploidy-induced G1 arrest.

To investigate a role of cyclin D1 in the survival of tetraploid cells, we assessed the ability of DCB-generated tetraploid cells to form colonies (Figure 2C). Although untreated cyclin D1-overexpressing cells demonstrated comparable plating efficiencies (PE) to control cells, the colony forming potential after DCB treatment was significantly increased in cyclin D1-overexpressing tetraploid RPE cells (Figure 2D). Cyclin D1-overexpression after DCB treatment also provided a marked growth advantage, as shown by the accumulation of Venus-positive G2/M subpopulations (supplementary Figure S2d, available at *Annals of Oncology* online). Interestingly, although knockdown of either p53 or p21 increased the proliferation of tetraploid cells, as depletion of either abrogates the G1 checkpoint [13], overexpression of cyclin D1 did not have a significant additive effect, suggesting that it may function in a linear pathway with p53 and p21 (supplementary Figure S2d, available at *Annals of Oncology* online).

As mammalian cells express three members of the cyclin D family [15], we also generated RPE cells overexpressing cyclin D2 in order to investigate functional redundancy within the family (supplementary Figure S3a, available at *Annals of Oncology* online). As with cyclin D1-overexpressing RPEs, a large proportion of DCB-induced RPE-cyclin D2 tetraploid cells were able to overcome p53/p21-mediated arrest (supplementary Figure S3b and c, available at *Annals of Oncology* online). In addition, both cyclin D1 and cyclin D2-overexpressing cells survived better than control cells after DCB-induced tetraploidization (supplementary Figure S3d, available at *Annals of Oncology* online and Figure 2E). Interestingly, we found that the cyclin D-dependent survival advantage was specific to tetraploid cells, as cyclin D-overexpressing diploid cells showed negligible differences in colony formation, relative to control cells (Figure 2E). This was recapitulated in HCT116 cells, where cyclin D1 and cyclin D2-overexpression only increased the survival of spontaneously occurring tetraploid cells (supplementary Figure S3e and f, available at *Annals of Oncology* online).

Collectively, these data demonstrate that overexpression of D-type cyclins overrides tetraploidy-induced G1 arrest in *TP53* wild-type cells and provides a specific survival advantage in the tetraploid state.

### p53 and p21 are elevated in tetraploid clones

**Figure 3.**
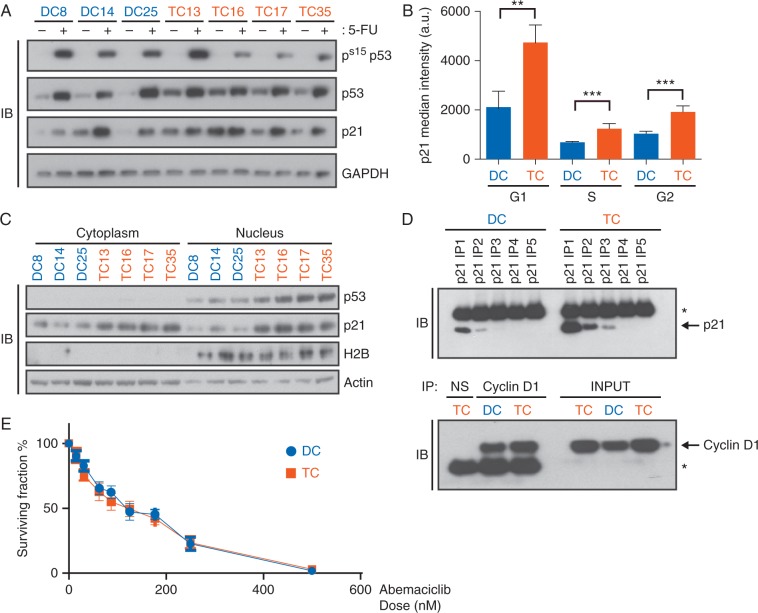
Mechanistic insights into the cyclin D-mediated tetraploidy tolerance. (A) Immunoblot (IB) of Serine 15 (S15) phosphorylated p53, total p53 and p21 protein levels in diploid (DC) and tetraploid (TC) HCT116 clones, after 5-FU treatment (5 μM; 16 h). (B) Graph demonstrating the median intensity of p21 in arbitrary units (a.u) in all phases of the cell cycle across the diploid and tetraploid clones. (C) Subcellular fractionation of HCT116 isogenic cells immunoblotted for p53 and p21 protein levels in diploid and tetraploid clones. H2B was used as a purity control and actin as a loading control. (D) Cell lysates from diploid and tetraploid clones were subjected to five rounds of immunodepletion with an anti-p21 antibody (IP1-5) and subsequently immunoprecipitated with anti-cyclin D1 antibody. Arrows indicate specific bands, asterisks indicate immunoglobulins cross-reacting with secondary antibodies. (E) Colony forming fraction of diploid and tetraploid clones after treatment with increasing doses of abemaciclib. Data shown as the average of three experiments, with three diploid and four tetraploid clones per data point in triplicate (errors bars are ±SEM).

In order to confirm functional p53 signalling, diploid and tetraploid HCT116 clones were treated with 5-FU, a member of the fluoropyrimidine compound family known to activate p53 [16]. Although there was variability in the response, all clones demonstrated elevated levels of p53, p53 serine-15 phosphorylation (Ser15) and induction of p21 after 5-FU treatment (Figure 3A), indicating a functional p53-mediated DNA damage response [17]. Interestingly, there was a significant increase in basal protein levels of both p53 and p21 in all tetraploid compared to diploid clones (Figure 3A and supplementary Figure S4a–c, available at *Annals of Oncology* online). Although the basal protein levels of p53 were increased, we were unable to detect p53-Ser15 phosphorylation in the absence of 5-FU (Figure 3A), suggesting that basal p53 accumulation in tetraploid cells was not DNA damage-induced.

Consistent with post-translational regulation of p53 [17], basal p53 mRNA levels remained largely unchanged between diploid and tetraploid clones (supplementary Figure S4d and e, available at *Annals of Oncology* online). In contrast, p21 mRNA levels were generally elevated in the tetraploid clones (supplementary Figure S4f and g, available at *Annals of Oncology* online) and consistent with p21 being a transcriptional target of p53 [17], siRNA-mediated p53-knockdown eliminated p21 expression (supplementary Figure S4h, available at *Annals of Oncology* online), confirming that p21 expression is driven by p53 in this system. Similarly, during acute tetraploidization in the RPE-FUCCI cell system, p53 and p21 levels followed a time-dependent increase in response to DCB treatment (supplementary Figure S4i, available at *Annals of Oncology* online).

As HCT116 tetraploid clones exhibited high basal levels of p53 and p21, a phenotype associated with cell cycle arrest in G1 [2], we investigated whether tetraploid clones exhibited distinct cell cycle kinetics, compared to diploid clones, using a nocodazole trap. Nocodazole prevents microtubule polymerization and spindle formation, leading to a G2/M arrest [18]. In the event of a p21-mediated G1 arrest, cells would be blocked from progressing to G2/M and, therefore, fewer cells would be ‘trapped’ by nocodazole. Untreated diploid and tetraploid clones were found to be cycling with comparable kinetics and there was no significant difference in the levels of G1 populations between the clones when subjected to the mitotic ‘trap’ (supplementary Figure S5a, available at *Annals of Oncology* online). In addition, downstream phosphorylation of pRb at Ser807/811, which takes place early during G1 by active cyclin D-CDK4/6 complexes and triggers G1/S progression [19], was similar between the diploid and tetraploid clones (supplementary Figure S5b and c, available at *Annals of Oncology* online). To exclude the possibility that p21 protein upregulation was unable to arrest cells by occurring in other cell cycle phases outside G1, we measured p21 levels by antibody-coupled flow cytometry. We found that tetraploid cells exhibited significantly higher levels of p21 compared to diploids throughout all stages of the cell cycle with the largest relative increase in G1 (Figure 3B).

Taken together, these data suggest that HCT116 tetraploid cells maintain normal cell cycle kinetics and cyclin D-CDK4/6 mediated Rb phosphorylation despite elevated p53 and p21 in a genome-doubled state.

### Cyclin D1-overexpression permits proliferation of genome-doubled cells through CDK-independent sequestration of p21

The normal cell cycle kinetics of tetraploid cells prompted us to investigate how this could be achieved in the presence of high levels of both p53 and p21. The anti-proliferative activity of p53 can be inhibited by cytoplasmic sequestration [17]. Similarly, p21 can also be sequestered in the cytoplasm, where it inhibits cytoplasmic pro-apoptotic regulators instead of nuclear CDK2, therefore promoting proliferation [20]. Subcellular fractionation of HCT116 clones demonstrated no specific enrichment of cytoplasmic p53 or p21 in the tetraploid clones (Figure 3C), suggesting that neither of these proteins were being sequestered in the cytoplasm.

A non-catalytic role of cyclin D1 involves the sequestration and, subsequently, inhibition p21 [15,21,22]. As p21 basal levels were elevated in cyclin D-overexpressing tetraploid clones (Figure 3A and supplementary Figure S4a, available at *Annals of Oncology* online), we speculated that cyclin D1-overexpression could mediate tetraploidy tolerance by sequestering p21 in cyclin D/p21 complexes with cyclin D in excess, counteracting the growth-inhibitory effects of p21. We tested this hypothesis by depleting p21 with multiple rounds of sequential immunoprecipitations in diploid (DC14) and tetraploid (TC13) lysates. Under these conditions, we found cyclin D1 to be in excess of p21, as high levels of cyclin D1 were still immunoprecipitated from lysates depleted of p21 (Figure 3D).

Currently, multiple CDK4/6 inhibitors are in phase III clinical trials [23] and cyclin D-overexpressing tumours have been suggested to be a potential selective biomarker [15]. In order to gain further mechanistic insights of the tolerance mechanism of cyclin D-overexpressing cells, we performed colony forming assays in the presence of increasing doses of the CDK4/6 inhibitor, abemaciclib. All diploid and tetraploid clones were equally sensitive to the drug, indicating that high cyclin D1 expression levels in tetraploid cells did not did not confer increased sensitivity to CDK4/6 inhibition (Figure 3E).

Taken together, these data suggest that cyclin D1-overexpressing cells overcome the inhibitory effects of p21, via a stoichiometric effect mediated by the ability of cyclin D to allow cell cycle progression in the presence of elevated p21, and not through a direct effect to the kinase activity of the cyclin D–CDK4/6 complex.

### TCGA analysis of D-type cyclin expression in genome-doubled tumours

In order to explore if the relationship between D-type cyclins and the p53/p21 axis is reflected *in vivo*, we investigated the TCGA dataset.

In *TP53* wild-type tumours, D-type cyclin and p21 expression did not generally correlate with either genome-doubling (supplementary Figure S6, available at *Annals of Oncology* online) or with genome stability (supplementary Figure S7, available at *Annals of Oncology* online), as measured by wGII score [9]. As late passage HCT116 tetraploid clones did not express the high levels of cyclin D1 and p21 observed in the early clones (Figure 1E and supplementary Figure S1d, available at *Annals of Oncology* online), it is possible that tumours which have undergone genome-doubling during their evolutionary history more closely resemble these later passage clones, and that high cyclin D1 expression is transient and dispensable with time.

**Figure 4.**
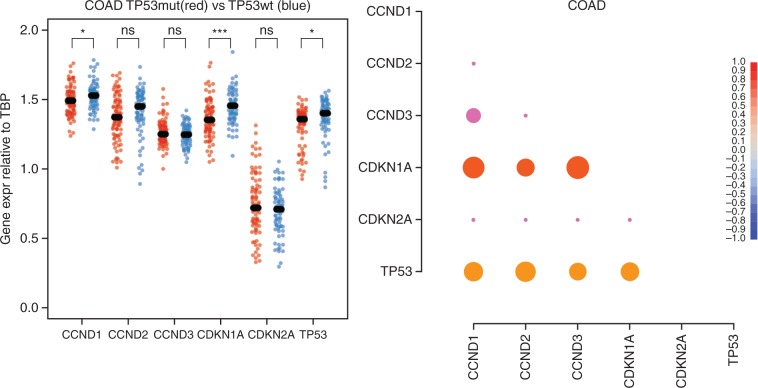
D-type cyclin expression in tumours. (A) Correlation of gene expression of D-type cyclins (CCND1-3) and components of the G1/S, p53-dependent checkpoint (CDKN1A, CDKN2A, *TP53*) with *TP53* mutant (red) versus *TP53* wild-type (blue) tumours in colorectal adenocarcinomas (COAD). Gene expression was normalized to TBP expression. (B) Spearman rank correlation analysis of gene expression of D-type cyclins and components of the G1/S p53-depenent checkpoint in *TP53* wild-type colorectal adenocarcinoma. The colour range indicated reflects the correlation values and the size of circles indicates *P* values, with larger circles representing smaller *P* values.

Nevertheless, gene expression analysis in colorectal adenocarcinomas (COAD) revealed that the expression levels of p21 and cyclin D1 were significantly higher in *TP53* wild-type compared to *TP53* mutant tumours (Figure 4A) and at least one D-type cyclin was significantly overexpressed in most other tumour types (supplementary Figure S8, available at *Annals of Oncology* online). Also, the correlation between the expression levels of p21 (CDKN1A), D-type cyclins (CCDN1-3) and p53 (*TP53*) was significant in *TP53* wild-type, colorectal adenocarcinoma (Figure 4B) and was maintained in other tumours types, for at least one D-type cyclin (supplementary Figure S9, available at *Annals of Oncology* online).

## Discussion

The importance of the p53 pathway in preventing tetraploidization has been well documented *in vitro* [4, 5] and more recently *in vivo* [13], in a variety of chemically induced and spontaneously arising tetraploid systems. Although mutations in *TP53* are significantly associated with genome-doubling in tumours [6, 7], we show that a large proportion of genome-doubled tumours have a functional p53 axis and no other known genetic aberrations that could permit tetraploidization.

We chose to investigate differences between naturally occurring HCT116 tetraploids and their isogenic diploid counterparts by quantitative proteomics. Our analysis identified cyclin D1 to be specifically elevated in tetraploid clones both at the protein and mRNA level. Interestingly, during the preparation of our article, transcriptome analysis was used to identify cyclin D2 as a facilitator of adaptation to genome-doubling, where both mRNA and protein levels were elevated in tetraploid cells [24]. We propose that although mRNA levels could follow a dosage-dependent regulation, the expression of specific genes such as the cyclin D family, involved in tetraploidization, might be selected for resulting in elevated protein levels and tetraploidy tolerance.

Here, using a combination of adapted and acute tetraploidization systems, we show that both cyclin D1 and D2 can confer a proliferative and survival advantage specifically to tetraploid cells, demonstrating the important role of D-type cyclins in tetraploidization. These findings are also consistent with recent data that demonstrate that tetraploid cells are more sensitive to depletion of cyclin D2 than diploid cells [24]. Our findings are recapitulated in tumours, where high expression of D-type cyclins is strongly associated with *TP53* wild-type tumours.

We show that spontaneously formed tetraploid clones are characterized by elevated p53, p21 and cyclin D1 protein levels but resist p21-mediated arrest, suggesting that the inhibitory effects of this CDK inhibitor are suppressed. Previous studies have demonstrated that cyclin D1 binds and sequesters p21, thus allowing progression from G1 to S phase [22, 25]. Consistently, in the isogenic HCT116 system, cyclin D1 protein levels are in excess of p21, suggesting that p21 is sequestered stoichiometrically.

As overexpression of D-type cyclins is sufficient to allow tetraploidization in our acute chemically induced system, we propose that increased D-type cyclin expression is an important licensing event occurring early during the tetraploidization process. Tumour data shows that *TP53* wild-type tumours exhibit significantly higher D-type cyclin and p21 expression levels, which also correlate strongly with each other, compared to *TP53* mutant tumours. We speculate that high cyclin D levels are needed to counter the elevated levels of p21, which are induced by the functional p53 axis in these tumours. However, our isogenic HCT116 system offers a unique advantage to study tetraploidization longitudinally and indeed, comparing late to early tetraploid clones, we find that not only do cyclin D1 protein levels specifically decrease to diploid levels in late clones, but this is also apparent for p21. Based on these findings, we propose that the functions of D-type cyclins comprise an acute response to tetraploidization, which could explain why D-type cyclin expression is not found to correlate with either genome-doubled or genetically unstable *TP53* wild-type tumours.

Finally, we demonstrate that tetraploid cells do not exhibit enhanced sensitivity to CDK4/6 inhibition, compared to diploid cells, suggesting that tetraploids are not ‘addicted’ to the catalytic activity of active CDK4/6 complexes. D-type cyclins are overexpressed in various cancer types and several studies are focusing on CDK4/6 inhibition to target these tumours [15, 23]. Data emerging from our analysis question specifically targeting D-type cyclin-overexpressing tetraploid tumours as an attractive therapeutic strategy.

Based on the work presented here, we propose a model for the development of genome-doubled tumours from a normal diploid precursor on a *TP53* wild-type background, where the first step would select for a cycling diploid cell with high cyclin D-expression. In the event of genome-doubling, for instance by failed cytokinesis, selection of a rare D-type cyclin-overexpressing cell could override p53-induced, p21-dependent G1 arrest by sequestering p21. The newly formed tetraploid cells would subsequently be allowed to enter S phase and complete mitosis. It is tempting to speculate that targeting the interaction between cyclin D1 and p21 through a protein–protein interaction small molecule inhibitor, an area that has recently seen substantial advances [26], may increase the levels of unbound p21, which in turn could block G1/S transition and promote cell cycle arrest and propagation of tetraploid cells.

## Supplementary Material

Supplementary Data
