# Supplementary material for: Cyclin D mediates tolerance of genome-doubling in cancers with functional p53
Source: Ann Oncol. 2016 Nov 17;28(1):149–56. doi: 10.1093/annonc/mdw612 (PMC5391719; doi:10.1093/annonc/mdw612)
Supplement: Supplementary Data [file mdw612_supp.zip › Crockford et al_Materials and Methods.docx]

**Materials and Methods**

### Whole Exome Sequencing (WES)

DNA extraction was carried out using the Qiagen DNeasy Blood and Tissue extraction kit, following the manufacturer’s instructions. DNA quality was assessed using a QuBit instrument (Life Technologies). Paired-end sequencing was carried out as described (1). Raw reads were aligned to the human hg19 genome assembly using BWA 0.5.9 (2) and post-processed to remove reads that mapped to multiple genomic loci and those that could have arisen from PCR duplication (using Picard tools 1.81; Picard). The Genome Analysis ToolKit (3) was used to perform indel realignment and indel left alignment. After filtering, a median exome coverage of 76X was observed per sample. Base-level nucleotide counts were obtained using the deepSNV package (4). Variant annotation was performed with ANNOVAR release 2012Oct23 (5). Intronic and synonymous mutations were removed from the total number of mutations, resulting in 4,138 non-synonymous SNVs. These mutations were observed in 3,287 genes. Mutated genes present in HCT-116 parental cells alone or present in all clones were removed, leaving 2,360 genes. Genes were subjected to hierarchical clustering, with genes on the Y-axis and samples on the X-axis.

### siRNA transfections

All small interfering RNA (siRNA, Dharmacon, Thermo Scientific) experiments were performed at 40nM final concentration using RNAiMax (Thermo Fisher) according to manufacturer’s instructions.

### Protein extraction, Immunoprecipitation and Immunoblotting

Cells were grown until 60-80% confluent, washed in PBS and trypsinised. Cells were pelleted, washed with PBS and re-suspended in cell lysis buffer (20mM Tris-HCl, 160mM sodium chloride, 1mM sodium EDTA, 1mM EGTA, 10 % (v/v) glycerol, 1% Triton-X-100, 10mM Beta glycerophosphate, 0.2mM sodium orthovanadate, 50mM sodium fluoride), supplemented with a mix of protease inhibitors (Roche). The lysates were cleared by centrifugation and the protein content was quantified using Bradford (Thermo Fisher). After electrophoresis and blotting, membranes were blocked in 5% non-fat milk in TBS-T (Tris-Buffered-Saline-Tween: 50mM Tris-Cl, pH 7.5. 150mM NaCl, 0.1% tween), and incubated with the indicated antibodies. After incubation with the appropriate HRP-conjugated secondary antibody (Dako) signal was detected with Immobilon Chemiluminescent HRP substrate (Merck Millipore). Quantification of protein bands was performed by analysis with Image J. For immune-depletion experiments, lysates were subjected to five rounds of sequential immunodepletion by addition of 1μg of p21 antibody and 30μl of protein A/G bead slurry per round. Immunodepletion was confirmed by immunoblotting with p21 antibody and the depleted lysates were subjected to cyclin D1 immunoprecipitation with 1 μg of cyclin D1 antibody. Cyclin D1 levels were detected by immunoblotting, as above. Subcellular fractions were performed as previously described previously (6). The antibodies used were: Cyclin D1 (556470) from BD Biosciences; p53 (sc-126), cyclin A1 (sc-271682), cyclin B1 (sc-752), cyclin E1 (sc-247), H2B (sc-8652) from Santa Cruz Biotechnology; p53 S15 (9248), p21 (2947), pRb (9309), pRb S807/811 (9308) from Cell Signalling Technology; Actin (ab49900), GAPDH (ab9385) from Abcam; HA from Crick Antibody Production.

### Quantitative PCR

RNA was extracted (RNeasy, Qiagen) and cDNA synthesis was performed using the first-strand cDNA synthesis kit (GE healthcare). Real-time quantitative polymerase chain reaction (qPCR) primers were designed using Primer-BLAST (7). QPCR was performed in triplicate on an Applied Biosystems 7900 real time PCR machine using SYBR® GreenER with premixed ROX (Invitrogen) according to the ΔΔCT method. Values were first normalised to GAPDH, before secondary normalisation to the parental HCT116 values.

### Antibody-coupled FACS

Cycling cells were trypsinised, washed and pelleted before fixation with 2% formaldehyde. Cells were then washed twice in 0.1% BSA, before blocking in the same solution for one hour at room temperature. After this time, cells were washed with blocking buffer and 0.5% Triton X-100 (Sigma-Aldrich) to permeabilise the cell nuclei, before a final wash in blocking buffer. At this time, cell pellets were incubated for 16 hours with 100μl of blocking buffer containing primary anti-p21. After a wash in blocking buffer, stained cells and unstained control cells were incubated in 100μl of buffer containing DAPI (1μg/ml) and the fluorescent secondary antibody (1:500) for one hour at room temperature. Staines cells were finally washed twice, resuspended in PBS and analysed on a Fortessa flow cytometer (BD Biosciences). Data was analysed with the FlowJo software.

### Nocodazole trap assay

Cells were seeded into 10cm dishes and treated with nocodazole (1μM), for 16 hours. After this period, cells were harvested and washed with PBS before fixing with 70% ethanol and stored at 4^o^C for 24 hours. Fixed cells were then treatment with RNase A and stained with propidium iodide, followed by DNA profile analysis on Fortessa flow cytometer.

### SILAC (extended)

Unlabelled, hydrochloride forms of L-arginine and L-lysine (R0K0) obtained from Sigma-Aldrich (light isotopes). Hydrochloride forms of L-arginine [13C6, 15N4] and L-lysine [13C6, 15N2] (R10K8) were obtained from CK Isotopes (heavy isotopes). After cells were passaged for a minimum of eight population doublings, 3 x10^6^ cells were lysed in 300 μl 8 M urea/Tris pH 8.5 on ice and sonicated for 10 seconds. After SDS-PAGE separation, the excised protein gel pieces were placed in individual wells of 96-well microtiter plates and de-stained with 50 % acetonitrile, 50 mM ammonium bicarbonate, reduced with 10 mM DTT and alkylated with 55 mM iodoacetamide (all reagents from Sigma-Aldrich). After alkylation, the proteins were digested with 6 ng/μl trypsin overnight at 37°C. The resulting peptides were extracted in 2 % formic acid, 1 % acetonitrile. For MS analysis, peptides were re-suspended in 0.1 %TFA and loaded on 50-cm Easy Spray PepMap column (Thermo Fisher Scientific). Reverse phase chromatography was performed using the RSLC nano U3000 (Thermo Fisher Scientific) with a binary buffer system at a flow rate of 250 nl/min. The in-gel digested samples were run on a linear gradient of solvent B (2- 40 %) in 144 minutes, total run time of 180 minutes including column conditioning. The nanoLC was coupled to a Q Exactive mass spectrometer using an EasySpray nano source (Thermo Fisher Scientific).

The percentage of incorporation of the heavy (R10K8) amino acids was assessed after the cells have doubled six times in the heavy medium. The cells were lysed and separated on SDS-PAGE and analysed by LC MS/MS, as described previously. The data was analysed using MaxQuant/Andromeda, but an additional label for heavy L-proline was established to assess the percentage of conversion of heavy L-arginine to heavy L-proline. Ratios of MaxQuant-derived intensities for peptides containing R10K8 versus R0K0 peptides were used to determine the percentage of incorporation (R10K8/ R0K0). The same strategy was used to calculate the level of L-arginine->L-proline conversion (R10K8P6/R10K8P0). Raw data files were analysed with MaxQuant software (version 1.3.0.5), as described previously (9).

### Viral overexpression in HCT116 and RPE-FUCCI

pLVX control, pLVX-cyclin D1-HA and pLVX-cyclin D2-HA lentivirus particles were generated as previously reported. Briefly, 293FT cells were plated at a density of 6x10^6^ and cultured for 16 hour. Cells were transfected and 24 hours after transfection, viral supernatants were harvested. Lentiviruses were transduced into cells in the presence of 8μg/ml polybrene. Stable populations were identified by puromycin selection.

### Cytokinesis failure - DCB assay

RPE-FUCCI cells were grown in 15cm tissue cultures dishes for 24 hours, after which cells were treated with 2 μM dihydrocytochalasin-B (DCB, Sigma-Aldrich) for a further 16 hours. Cells were then extensively washed in PBS, allowed to recover in fresh media for one hour before trypsinisation and DNA staining with 10μg/ml Hoescht in PBS for one hour at 37^o^C. Samples were sorted using an Influx BD cell sorter (BD Biosciences), by used of the FUCCI markers, excited at 561 (mCherry emission 610/20) and 488 (Venus emission 530/40), and 405 (Hoechst emission 460/50). mCherry^+ve^/Venus^–ve^ G1 tetraploid cells were sorted and kept on ice before washing and seeding for downstream applications. Unsorted RPE-FUCCI cells were used as controls. In other experiments, as indicated, control vector or cyclin D1, cyclin D2-overexpressing RPE-FUCCI cells were subjected to the above treatment and sorted, by use of FUCCI markers and Hoechst, into 2N (diploids in G1) or 4N mCherry^+ve^/Venus^–ve^ (tetraploids in G1) and seeded into clonogenic assays.

To assess the proliferative potential conferred by D-type cyclins, cells were treated with DCB as above and subsequently trapped in G2/M by treatment with S-Trityl-L cysteine (STLC), an inhibitor of Eg5 which arrests cells in anaphase (8), for 12 hours. After 72 hours, plates were fixed with 4% paraformaldehyde (PFA) and stored at 4^o^C, washed with PBS, permeabilised with 0.5% Triton X-100 for five minutes at room temperature and stained with DAPI (1μg/ml). Plates were imaged using the Cellomics Arrayscan Vti machine with a 10X0.3 numerical aperture objective. The percentage of G2/M cells was calculated using the Target Activation Cellomics Bioapplication (TACB).

Reference list

1. Gerlinger M, Rowan AJ, Horswell S, Larkin J, D E, Gronroos E, et al. Intratumor heterogeneity and branched evolution revealed by cancer sequencing. New England Journal of Medicine *IN PRESS*. 2012 *in press*.

2. Li H, Durbin R. Fast and accurate short read alignment with Burrows-Wheeler transform. Bioinformatics. 2009;25(14):1754-60. doi: 10.1093/bioinformatics/btp324. PubMed PMID: 19451168; PubMed Central PMCID: PMCPMC2705234.

3. DePristo MA, Banks E, Poplin R, Garimella KV, Maguire JR, Hartl C, et al. A framework for variation discovery and genotyping using next-generation DNA sequencing data. Nat Genet. 2011;43(5):491-8. doi: 10.1038/ng.806. PubMed PMID: 21478889; PubMed Central PMCID: PMCPMC3083463.

4. Gerstung M, Beisel C, Rechsteiner M, Wild P, Schraml P, Moch H, et al. Reliable detection of subclonal single-nucleotide variants in tumour cell populations. Nat Commun. 2012;3:811. doi: 10.1038/ncomms1814. PubMed PMID: 22549840.

5. Wang K, Li M, Hakonarson H. ANNOVAR: functional annotation of genetic variants from high-throughput sequencing data. Nucleic Acids Res. 2010;38(16):e164. doi: 10.1093/nar/gkq603. PubMed PMID: 20601685; PubMed Central PMCID: PMCPMC2938201.

6. Schreiber E, Matthias P, Muller MM, Schaffner W. Rapid Detection of Octamer Binding-Proteins with Mini-Extracts, Prepared from a Small Number of Cells. Nucleic Acids Res. 1989;17(15):6419-. doi: DOI 10.1093/nar/17.15.6419. PubMed PMID: WOS:A1989AK53100072.

7. Ye J, Coulouris G, Zaretskaya I, Cutcutache I, Rozen S, Madden TL. Primer-BLAST: a tool to design target-specific primers for polymerase chain reaction. BMC bioinformatics. 2012;13:134. Epub 2012/06/20. doi: 10.1186/1471-2105-13-134. PubMed PMID: 22708584; PubMed Central PMCID: PMC3412702.

8. Skoufias DA, DeBonis S, Saoudi Y, Lebeau L, Crevel I, Cross R, et al. S-trityl-L-cysteine is a reversible, tight binding inhibitor of the human kinesin Eg5 that specifically blocks mitotic progression. The Journal of biological chemistry. 2006;281(26):17559-69. Epub 2006/03/02. doi: 10.1074/jbc.M511735200. PubMed PMID: 16507573.
