# Supplementary material for: Cyclin D mediates tolerance of genome-doubling in cancers with functional p53
Source: Ann Oncol. 2016 Nov 17;28(1):149–56. doi: 10.1093/annonc/mdw612 (PMC5391719; doi:10.1093/annonc/mdw612)
Supplement: Supplementary Data [file mdw612_supp.zip › Crockford et al_Supplementary Figure Legends.docx]

**Supplementary Figure Legends**

**Supplementary Figure S1**

(a) The proportion of genome-doubled (GD; red) versus non genome-doubled (nGD; blue) tumours is indicated for each cancer type. Lightly coloured bars depict TP53 and RB1 mutant tumours whereas darkly coloured one depict TP53 and RB1 wild-type. (b) SILAC correlation plot displaying two inversely-labelled replicate experiments. Late TC13 cells were labelled ‘light’ (L) on the horizontal axis and ‘heavy’ (H) on the vertical axis, while early DC14 cells were labelled inversely. In either case, ‘heavy’ species were divided by ‘light’ and ratios representing log2 fold differences between clones were plotted. Box plots showing the median (stripe), the 25th to 75th percentile (box) and outliers (open circles) for both replicate experiments. Cyclin-D1 is indicated. (c) Q-PCR quantification of cyclin D1 mRNA levels of diploids and tetraploid clones; values were normalised to GAPDH mRNA levels and expressed as a fold change relative to HCT116. (d) Quantification of cyclin D1 mRNA levels in pooled diploid and tetraploid clones. (e) Immunoblot (IB) of p21 levels in diploid and tetraploid, early and late clones, as indicated by the passage number. GAPDH was used as loading control.

**Supplementary Figure S2**

(a) Experimental procedure used to isolate tetraploid RPE cells using the FUCCI system. Cells were seeded before treatment with 2μM DCB for 16 hours to induce cytokinesis failure and tetraploidisation. The FUCCI system was used to separate 4N G1 tetraploid and G2 diploid cells by FACS sorting the mCherry-positive G1 tetraploid cells for downstream assays. (b) Immunoblot (IB) of cyclin D1 in RPE-FUCCI, control or cyclin D1-overexpressing cells; GAPDH was used as loading control. (c) DNA ploidy plots of RPE-FUCCI control and cyclin D1-overexpressing cells, indicated the basal tetraploid levels of cycling cells. (d) Percentage of surviving (G2/M) RPE-FUCCI cells 72 hours post-DCB (2μM) treatment, with p53 or p21 knockdown serving as positive controls, and untreated (UNT), mock treated (mock) and non-targeting siRNA (NT) negative controls.

**Supplementary Figure S3**

(a) Immunoblot of HA-tagged cyclin D1 and cyclin D2 in RPE-FUCCI, control or cyclin D1 or D2-overexpressing cells, as detected by HA antibody. Arrow indicates specific band and asterisk non-specific polypeptides. (b) FACS strategy used to isolate control, cyclin D1 or cyclin D2-overexpressing RPE-FUCCI cells. Cells were treated with DCB as in (Supplementary Figure S2a) and sorted two-ways in 2N G1 (diploid) and 4N mCherry-positive G1 (tetraploid). (c) DNA ploidy profiles of RPE-FUCCI cells, control or cyclin D1 or cyclin D2-overexpressing, treated as in (Supplementary Figure 2a). (d) Colony formation assays of RPE-FUCCI cells, control or cyclin D1 or cyclin D2-overexpressing, treated with DCB (2μM, 18h) and FACS-sorted into 2N G1 and 4N G1 cell populations. (e) FACS strategy used to isolate naturally-occuring diploid and tetraploid HCT116 cells, as indicated. (f) HCT116 control or cyclin D1 or cyclin D2-overexpressing cells were FACS-sorted and plated for clonogenic assays. Colony forming units from triplicates of two independent experiments were counted and data is presented as a fold change relative to HCT116 control cells.

**Supplementary Figure S4**

(a) Immunoblot (IB) of p53 and p21 protein levels in diploid (DC) and tetraploid (TC) HCT116 clones, as indicated. GAPDH serves as a loading control. (b) Quantification of p53 protein levels in pooled diploid and tetraploid clones. (c) Quantification of p21 protein levels in pooled diploid and tetraploid clones. (d) Quantification of p53 mRNA levels in all clones, normalised to GAPDH and presented relative to HCT116 levels. (e) Quantification of p53 mRNA levels in pooled diploid and tetraploid clones. (f) Quantification of p21 mRNA levels in all clones, normalised to GAPDH and presented relative to parental HCT116 levels. (g) Quantification of p21 mRNA levels in pooled diploid and tetraploid clones. (h) Immunoblot of p53 and p21 protein levels in diploid and tetraploid clones, under conditions of control or p53 knockdown. GAPDH serves as a loading control. (i) Immunoblot of p53, p21 and cyclin D1 protein levels in RPE-FUCCI, control or cyclin D1-overexpresssing cells, during a time-course treatment with DCB (2μM) for the indicated time. GAPDH serves as a loading control.

**Supplementary Figure S5**

(a) Graph demonstrating G1 phase fractions of diploid and tetraploid clones of cycling cells (untreated) or after treatment with nocodazole (NOC) in mitotic trap experiments. (b) Phosphorylated pRb-Ser807/811 and total pRb levels analysed by immunoblotting (IB) across diploid (DC) and tetraploid (TC) HCT116 clones; GAPDH was used as a loading control. (c) Quantification of phospho-pRb Ser807/11 levels relative to total pRb levels in pooled diploid and tetraploid clones.

**Supplementary Figure S6 Correlation of expression of D-type cyclins and components of the G1/S-p53 checkpoint with genome-doubling in *TP53* wild-type tumours**

Correlation of gene expression of D-type cyclins (CCND1-3) and components of the G1/S, p53-dependent checkpoint (CDKN1A, CDKN2A, *TP53*) with genome-doubled (red) versus non genome-doubled (blue) LUng ADenocarcinoma (LUAD), LUng Squamous cell Carcinoma (LUSC), BReast CAncer (BRCA), COlorectal ADenocarcinoma (COAD), SKin Cutaneous Melanoma (SKCM), Kidney Renal Clear cell Carcinoma (KIRC), Head-Neck Squamous cell Carcinoma (HNSC) and BLadder Cancer (BLCA) tumours.

**Supplementary Figure S7 Correlation of expression of D-type cyclins and components of the G1/S-p53 checkpoint with genome stability in genome-doubled *TP53* wild-type tumours.**

Correlation of high (red) vs low (blue) gene expression of D-type cyclins (CCND1-3) and components of the G1/S, p53-dependent checkpoint (CDKN1A, CDKN2A, *TP53*) with genome instability, as measured by wGII score, in *TP53* wild-type, genome-doubled LUng ADenocarcinoma (LUAD), LUng Squamous cell Carcinoma (LUSC), BReast CAncer (BRCA), COlorectal ADenocarcinoma (COAD), SKin Cutaneous Melanoma (SKCM), Kidney Renal Clear cell Carcinoma (KIRC), Head-Neck Squamous cell Carcinoma (HNSC) and BLadder Cancer (BLCA) tumours.

**Supplementary Figure S8 Correlation of D-type cyclin and p21 expression with p53 status across different tumour types**

Correlation of gene expression of D-type cyclins (CCND1-3) and components of the G1/S, p53-dependent checkpoint (CDKN1A, CDKN2A, *TP53*) with *TP53* mutant (red) versus *TP53* wild-type (blue) across genome-doubled LUng ADenocarcinoma (LUAD), LUng Squamous cell Carcinoma (LUSC), BReast CAncer (BRCA), SKin Cutaneous Melanoma (SKCM), Kidney Renal Clear cell Carcinoma (KIRC), Head-Neck Squamous cell Carcinoma (HNSC) and BLadder Cancer (BLCA) tumours.

**Supplementary Figure S9 Correlation of D-type cyclin and p21 expression in *TP53* wild-type genome-double tumours across different tumour types**

Spearman correlation analysis of gene expression of D-type cyclins and components of the G1/S p53-dependent checkpoint in *TP53* wild-type, genome-doubled LUng ADenocarcinoma (LUAD), LUng Squamous cell Carcinoma (LUSC), BReast CAncer (BRCA), SKin Cutaneous Melanoma (SKCM), Kidney Renal Clear cell Carcinoma (KIRC), Head-Neck Squamous cell Carcinoma (HNSC) and BLadder Cancer (BLCA) tumours. The colour range indicates Spearman correlation values and the size of circles indicates p-values, with larger circles representing smaller p-values.
