# Supplementary figures and images for: Cyclin D mediates tolerance of genome-doubling in cancers with functional p53
Source: Ann Oncol. 2016 Nov 17;28(1):149–56. doi: 10.1093/annonc/mdw612 (PMC5391719; doi:10.1093/annonc/mdw612)

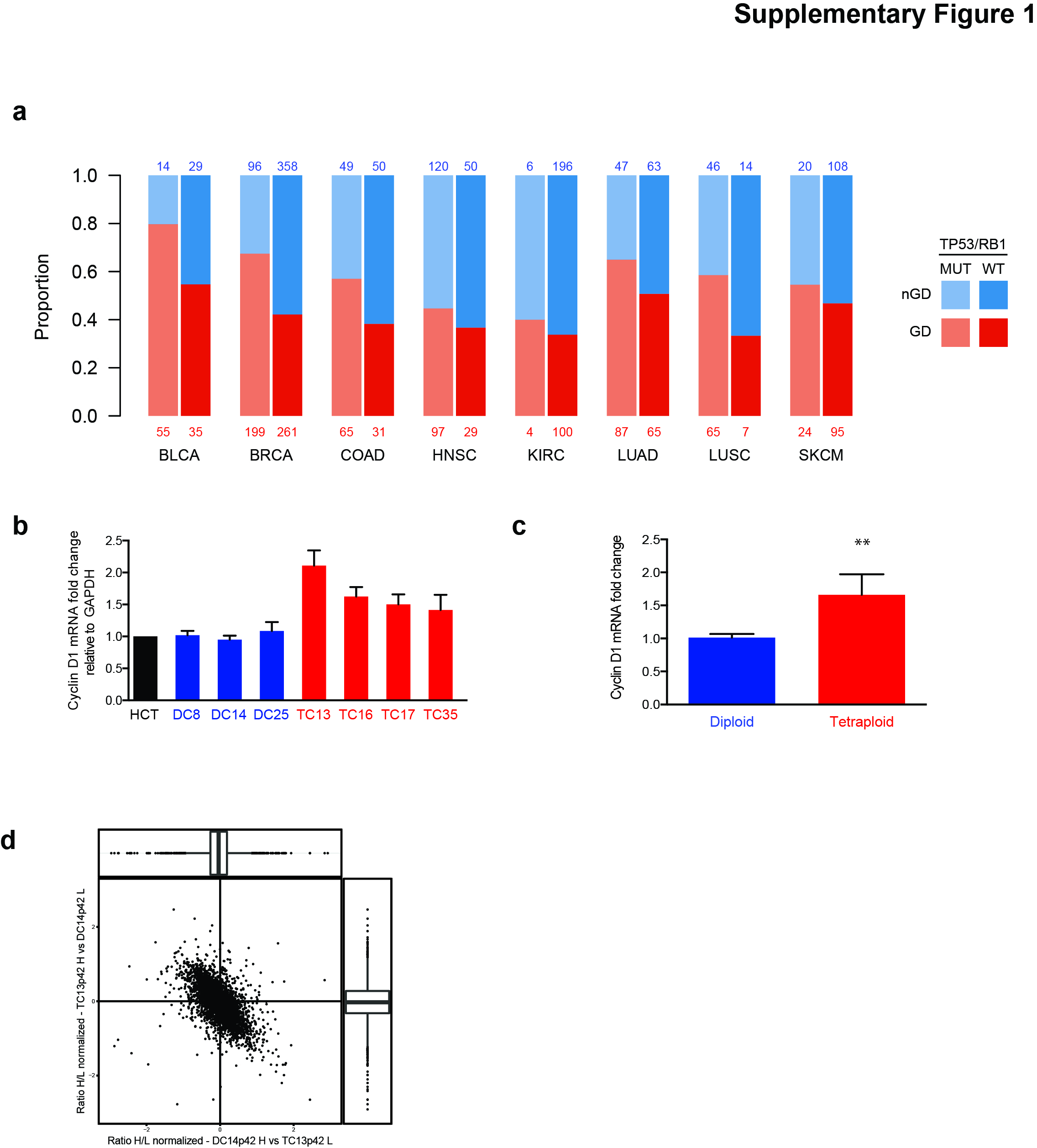

Supplement: Supplementary Data [file mdw612_supp.zip › Supp 1.jpg]

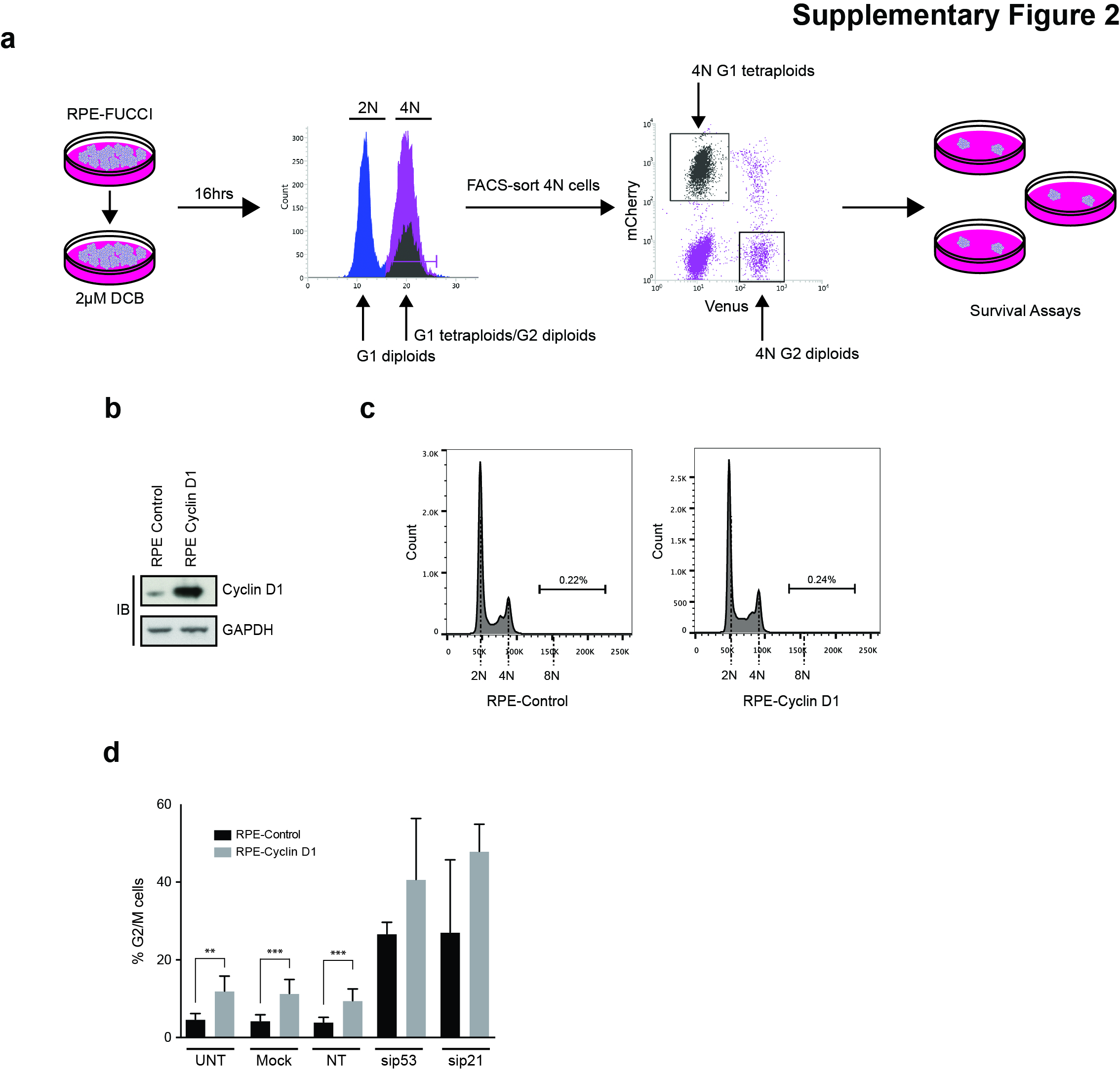

Supplement: Supplementary Data [file mdw612_supp.zip › Supp 2.jpg]

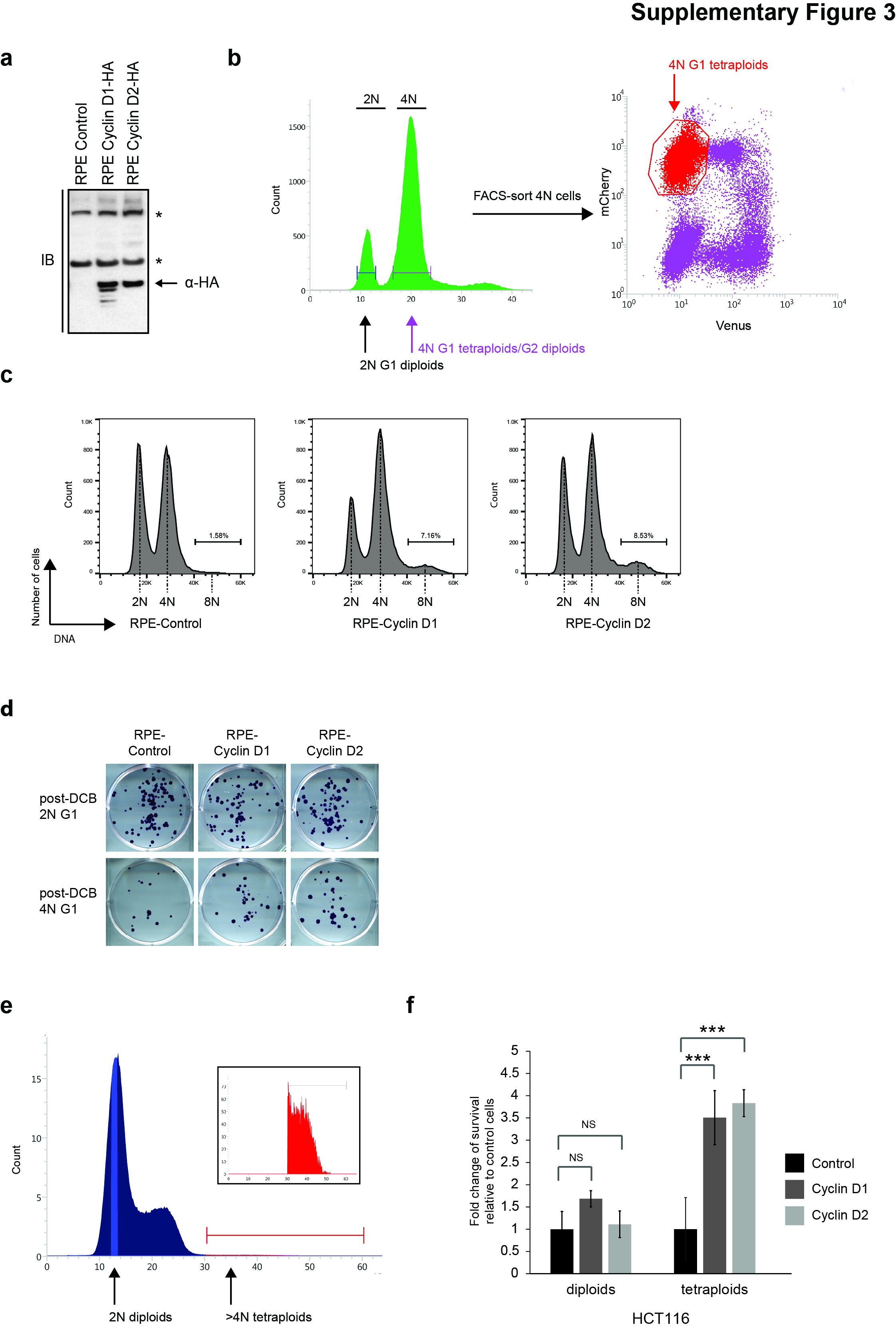

Supplement: Supplementary Data [file mdw612_supp.zip › Supp 3.jpg]

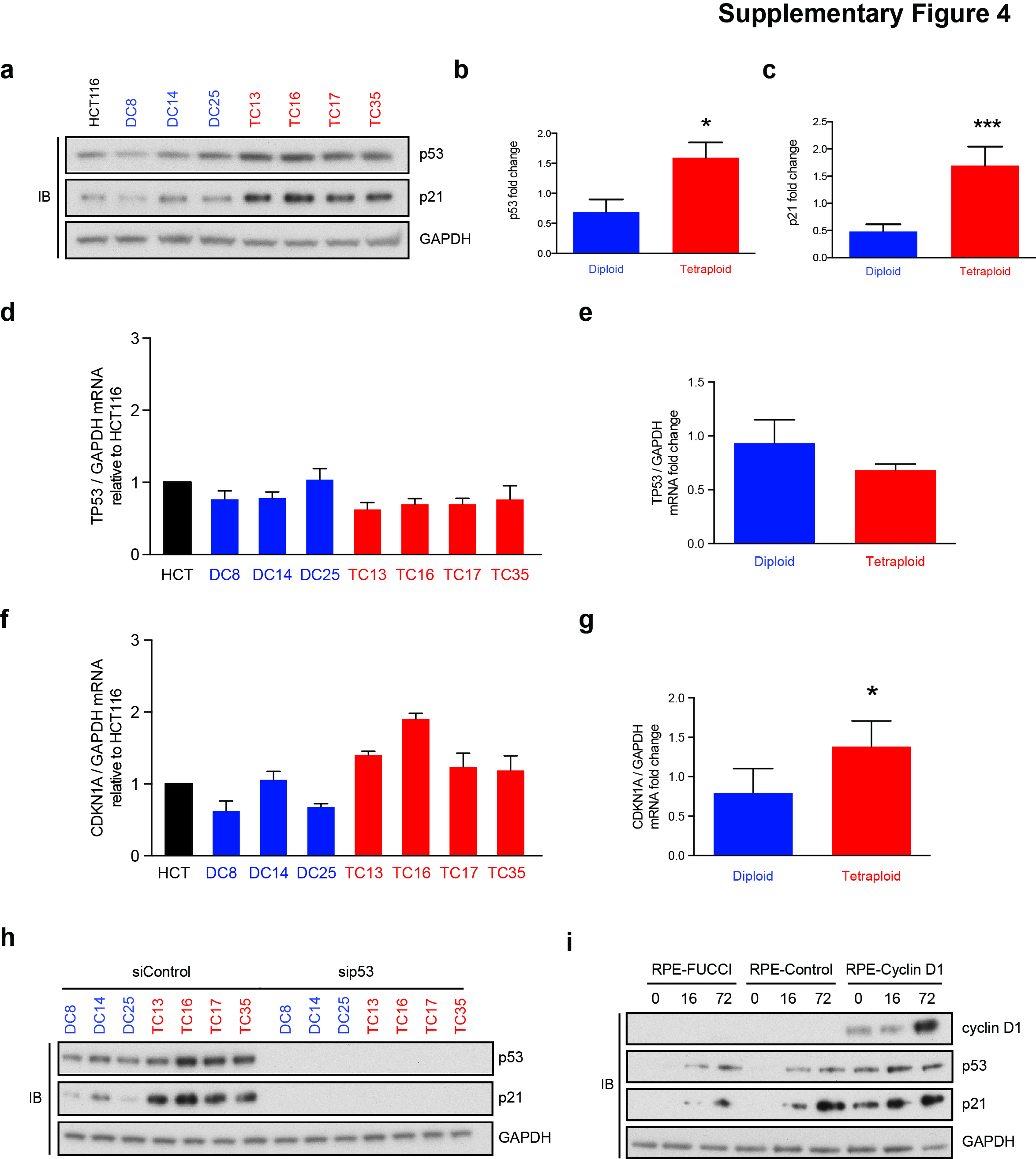

Supplement: Supplementary Data [file mdw612_supp.zip › Supp 4.jpg]

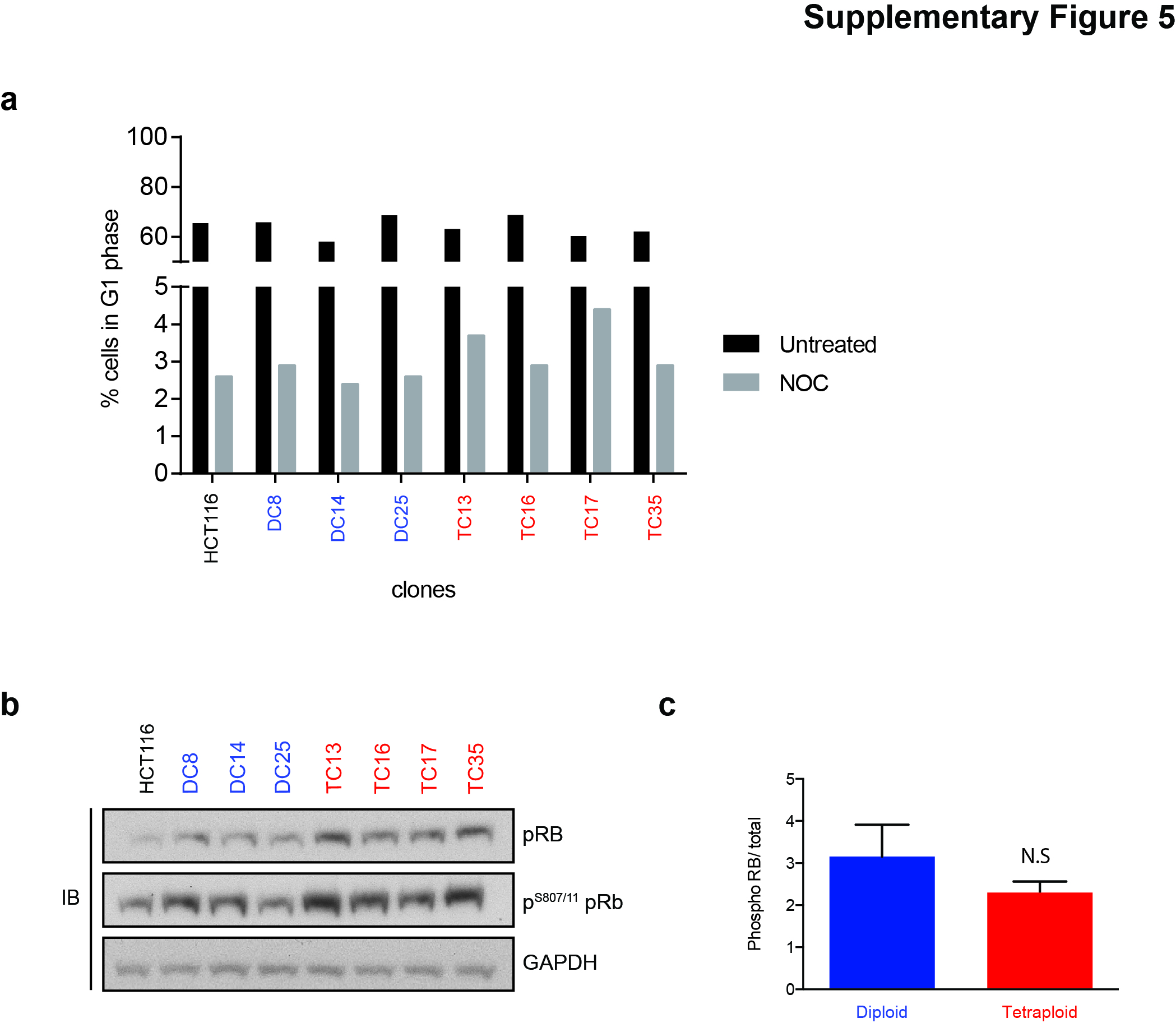

Supplement: Supplementary Data [file mdw612_supp.zip › Supp 5.jpg]

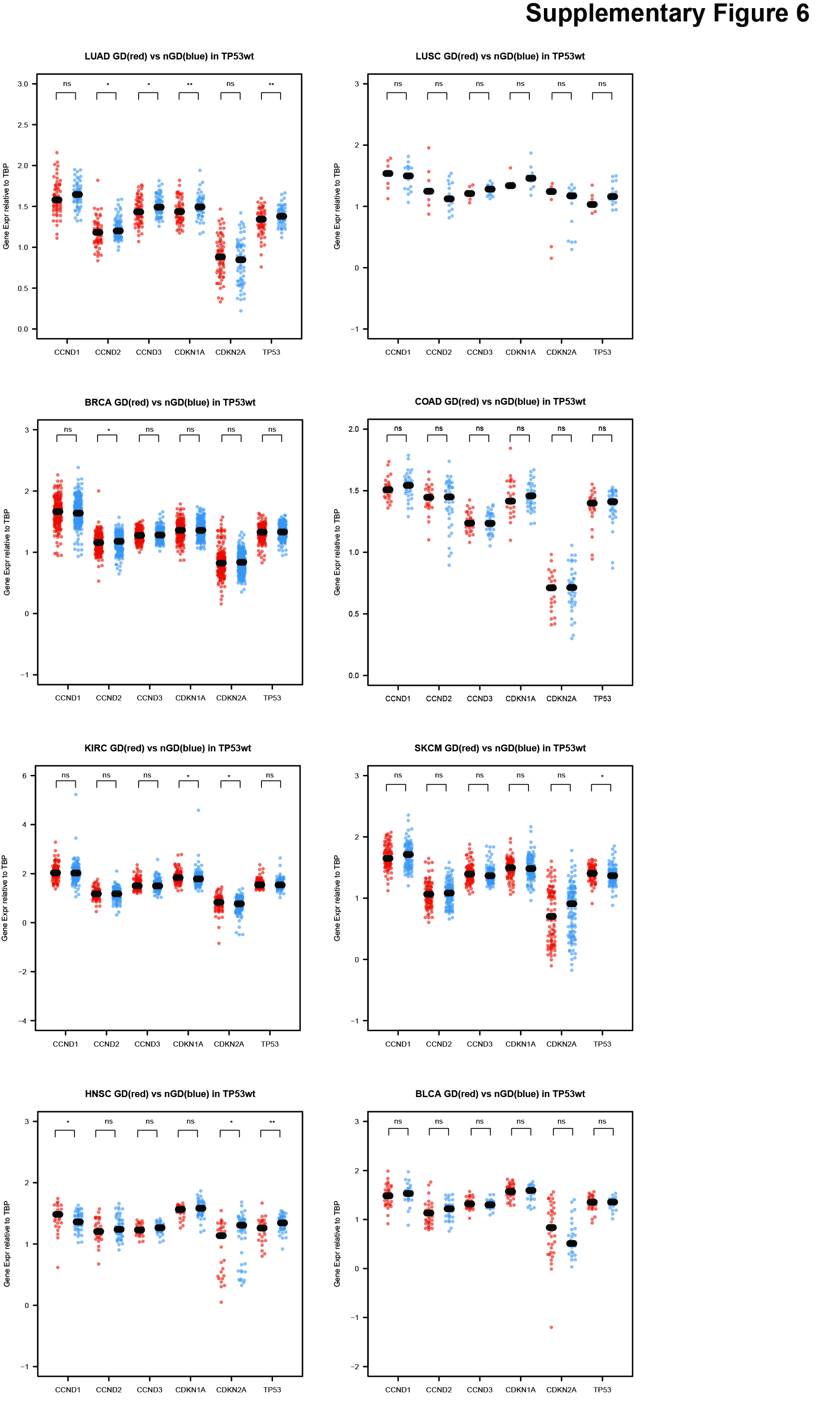

Supplement: Supplementary Data [file mdw612_supp.zip › Supp 6.jpg]

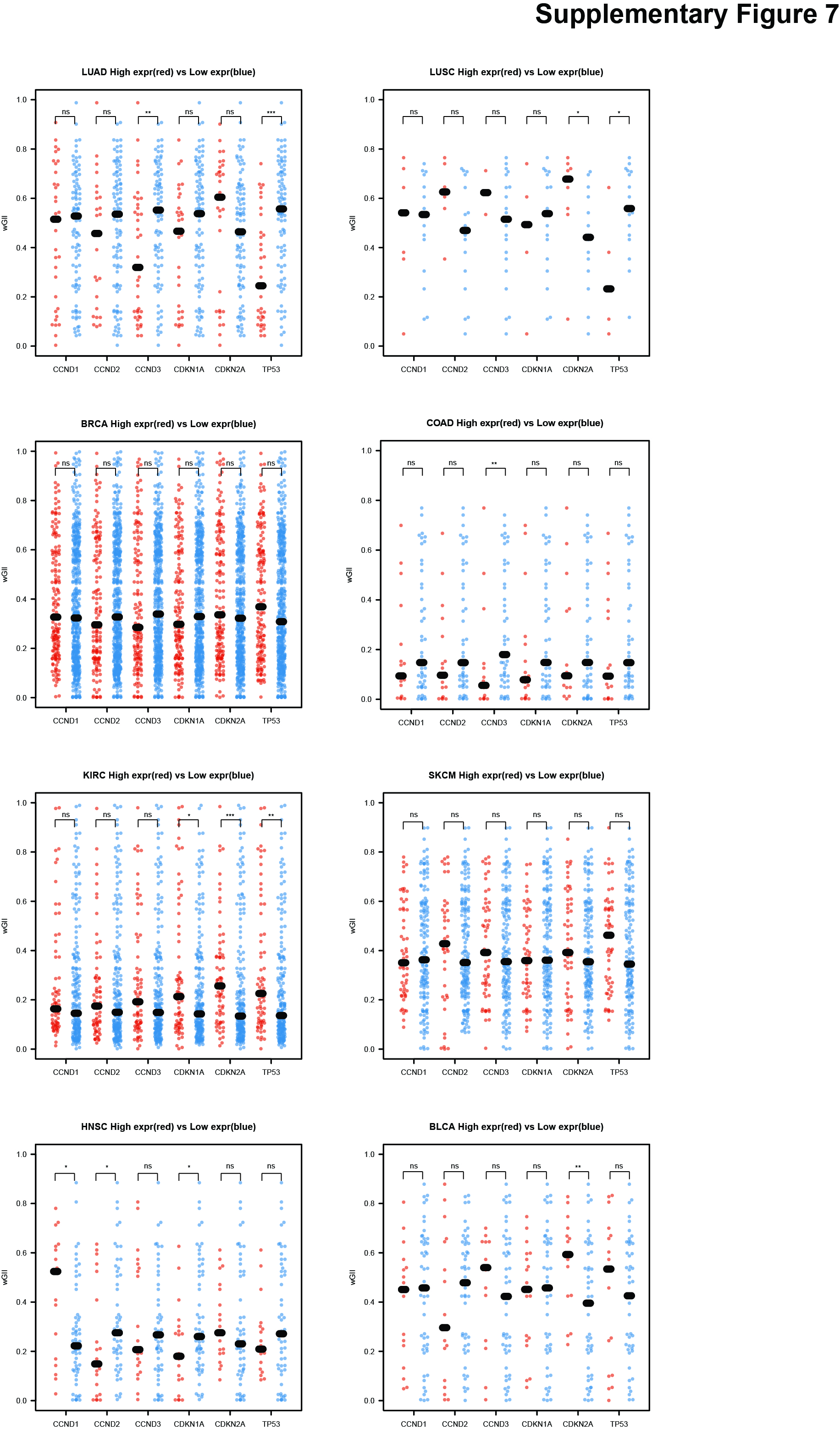

Supplement: Supplementary Data [file mdw612_supp.zip › Supp 7.jpg]

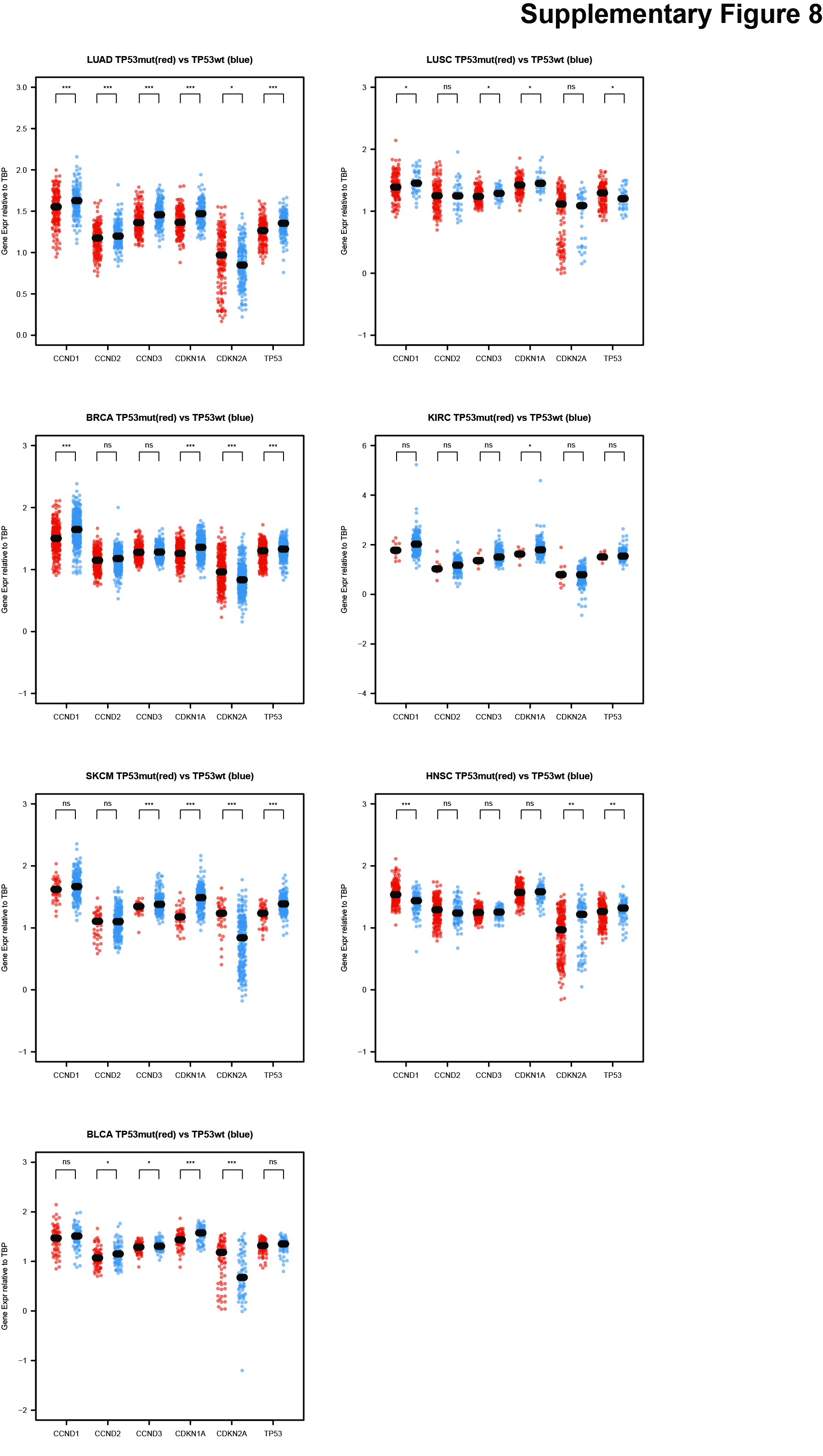

Supplement: Supplementary Data [file mdw612_supp.zip › Supp 8.jpg]

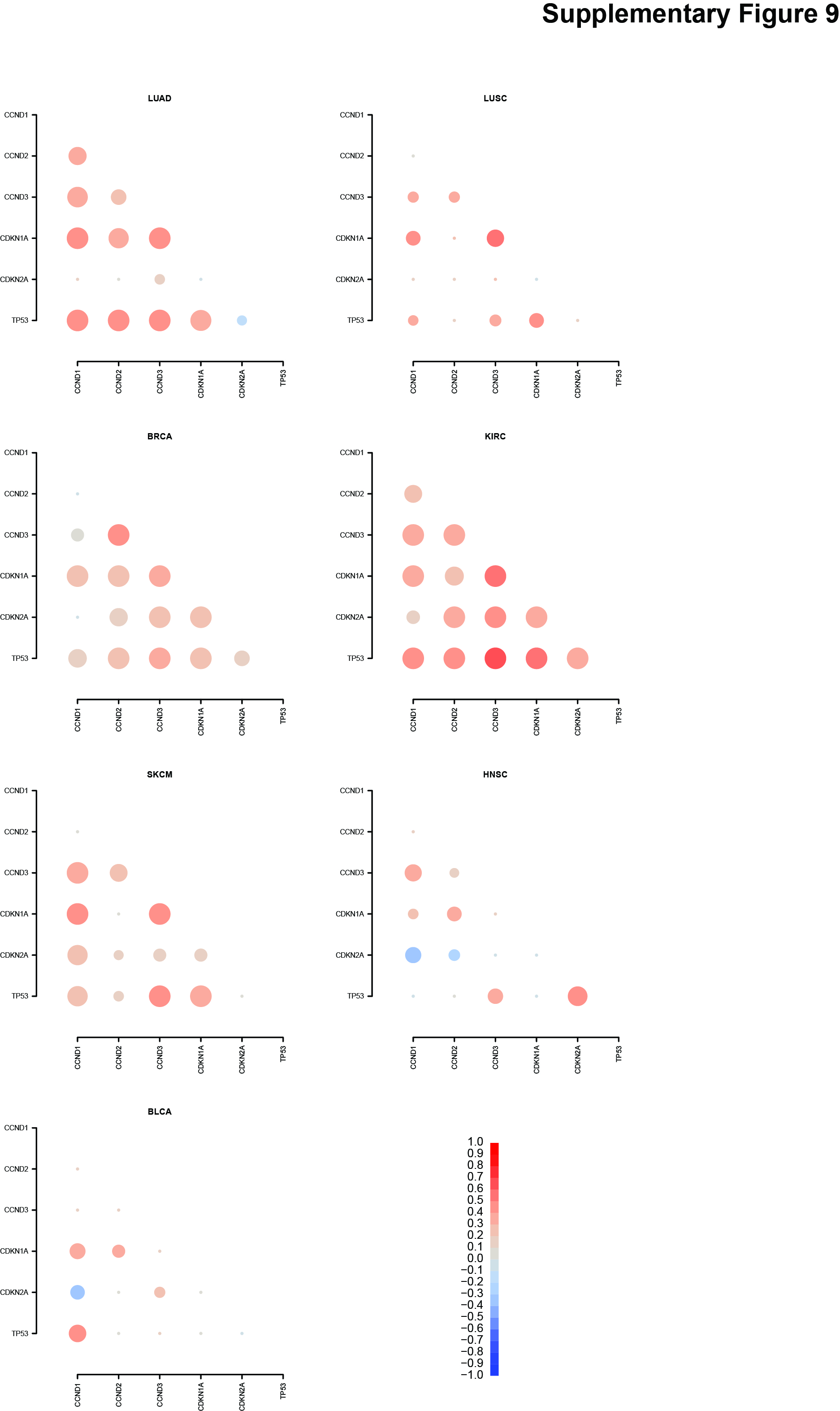

Supplement: Supplementary Data [file mdw612_supp.zip › Supp 9.jpg]
